# Reasons for non-participation in a self-care training program for diabetic patients: a qualitative study

**DOI:** 10.1186/s12913-022-07541-1

**Published:** 2022-01-29

**Authors:** Tahere Sharifi, Javad Javan-Noughabi, Zahra Asadi, Marzie Zarqi

**Affiliations:** 1 Department of Health Management and Economics, School of Health, Tehran University of Medical Sciences, Tehran, Iran; 2 Social Determinants of Health Research Center, Mashhad University of Medical Sciences, Mashhad, Iran; 3 Department of Health Economics and Management Sciences, School of Health, Mashhad University of Medical Sciences, Mashhad, Iran; 4 Department of Change Management, Faculty of Economics and Administrative Sciences, Ferdowsi University, Mashhad, Iran

**Keywords:** Diabetes, Self-care training, Qualitative study

## Abstract

**Introduction:**

Self-care behaviors in diabetic patients is considered an important factor for controlling the diabetes. Therefore, diabetic patients need training the self-care behaviors to control this disease. This study aims to investigate the reasons for diabetic patients’ non-participation in a self-care training program.

**Method:**

This qualitative study was carried out between 1 April to 1 July 2019. We used in-depth semi-structured interviews with 30 diabetic patients who did not participate in the self-care training program. Data analysis was conducted using content analysis with MAXQDA software.

**Findings:**

The results of this study showed that there are 5 themes and 14 sub-themes for patients’ non-participation in self-care training program. Themes included access; individual, familial and social factors; attitude and awareness; motivator factors and need Factors. Also sub-themes were physical access, time access, physical abilities, social-familial responsibilities, attitude to disease, attitude to education, attitude to health, awareness, incentive, communication, teaching methods, perceived risk, access to other educational resources and self-efficacy.

**Conclusion:**

According to the results of this study, simple physical and time access, offering high-quality education, providing virtual and distant training, organizing and designing modern training methods can lead to increase participation in self-care training programs.

## Introduction

Diabetes is among metabolic and multifactorial disorders characterized by high blood sugar or hyperglycemia, which is due to a disruption in insulin secretion or function, or both [1]. Diabetes is called a “dormant epidemic” and is a cause of the death of about 1.5 million people in the world [2]. According to statistics, the number of diabetic patients in 2000 was 171 m, and it is estimated to reach 578 m in 2030 from 463 m in 2019 [3]. In Iran, the national outbreak of 11.4% means that there are as many as 4 million mature diabetic patients; this figure is estimated to reach 9.2 million in 2030 [4]. International expenses of the condition are reckoned as high as 1.3 billion dollars including direct care and indirect expenses due to inability, disability and early deaths [5]. One of the most important factors in controlling this disease is the participation of patients in the treatment of the disease. Therefore, diabetic patients and their families need to learn about practices such as monitoring blood sugar, selecting a proper diet and increasing physical activities [6]. To this end, a simple concept called “empowerment in self-care” is posed, which means enhancing self-determination and self-adjustment [7]. Patients are encouraged to cooperate through sharing information and interaction in order to have maximum participation in their own treatment [7]. Teaching self-care behavior is effective in improving the quality of life and reducing expenses [7]. Although numerous studies have confirmed the effectiveness of self-care schemes, there are high levels of attrition in patients’ participation [8]. Those who abandon self-care plans to control diabetes suffer from worse health consequences [9]. Currently, there are little and discrepant information about factors related to non-participation in self-care schemes in diabetic patients [9]. It has been clarified that success or failure in any training scheme depends on individuals’ fundamental beliefs and attitudes [10]. Most studies conducted on self-care schemes assessed the effects of training programs on enabling and behavior change in diabetic patients [11–16]; few studies have been examined the effective factors in patients’ non-participation in self-care schemes from the point of view of those who have abandoned self-care schemes [10, 17]. However, both previous studies were conducted in developed countries (Canada and Germany) [10, 17]. This is while the situation for developing countries may be completely different. As far as we know, no study has been conducted on the reasons for not participating in self-care programs in Iran. Nevertheless, individuals themselves are a valuable source in understanding the disuse of health services, a fact usually ignored in studies. Regarding the money and time spent in planning and structuring self-care training schemes, discovering the reason why diabetic patients do not complete their self-care training program and abandon it halfway is warranted in order to achieve success in self-care schemes. Thus, this study seeks to investigate the reasons for diabetic patients’ non-participation in self-care training schemes.

## Methods

### Design

This was a qualitative study that aimed to assess obstacles to diabetic patients’ attending in self-care training schemes.

### Theoretical orientation

In the qualitative study, we seek deeper and more accurate information about the behavior of diabetic patients that could not be identified using a quantitative study. A qualitative approach was designated for this study because this method provides a deep understanding of the participant under investigation and practically was the only approach responding to the research questions as it is generally believed that the choice of the research method is determined by the research questions [18].

### Context

The prevalence of DM in Iranian adults aged 25–70 years was 11.9% in 2011, and it is predicted that in the year 2030 there will be about 9.2 million people with diabetes in Iran [4]. In 2015, 9309 deaths due to diabetes were registered in Iran and age standardized mortality rate associated with diabetes in Iran is increased from 8.7 in 2000 to 11.3 in 2015 [19]. There are three strategies to control diabetes in Iran. Reducing blood glucose to the recommended targets through lifestyle and pharmacotherapy; assessment and reduction of related cardiometabolic risk factors (e.g. overweight/obesity, hypertension, and dyslipidemia); and scheduled regular screening for micro- and macrovascular complications with prompt management of incident cases [20].

### Interventions: self-care training program

A self-care training program for diabetic patients was implemented in the Mashhad city, northeast Iran, between 1 April to 1 July 2019. Self-care training courses for diabetic patients in health centers lasted 3 months and involved 12 sessions. Self-care training program covered several main topics such as diet, physical activity, blood sugar monitoring and foot care.

### Participants

Among patients who did not participate in the training program, 30 individuals were purposefully selected for this study. Non-participation in the training program was defined as attendance at less than half of the sessions. The mean age of the participants in this study was 59.4 ± 9.39 years. The majority of participants were female (66.7%), married (80%), employment (86.7%), and with university education (60%). Socio-demographic characteristics are shown in Table 1.

**Table 1:** Socio-demographic characteristics of participants

Variable	N (%)
**Sex**	
**Male**	10 (33.3%)
**Female**	20 (66.7%)
**Marital status**	
**Married**	24 (80%)
**Single**	6 (20%)
**Education level**	
**University education**	18 (60%)
**Non-university education**	12 (40%)
**Employment status**	
**Employment**	26 (86.7%)
**Un-employment**	4 (13.3%)
**Age**	
**Mean ± SD**	
59.4 ± 9.39	

### Data collection

The required data were collected through in-depth semi-structured interviews. Interviews were conducted by the corresponding author (JJN), a faculty member of Mashhad University of Medical Sciences (MUMS) with experience in conducting qualitative interviews. Every interview began with a general question, “What are the main reasons for non-participation in the self-care training schemes?”. Then, the interviewees were asked to express their opinions, experiences, and views to achieve further information. Also, we used queries like “can you explain further?” or “what do you mean exactly by saying that” or “could you give us more examples?” The interviews continued until data saturation; that is, the new data entered into the study did not create new theme or change the existing themes. The data was saturated with 30 interviews each lasting 35 min on average. All interviews were conducted face to face and physical mode.

### Data analysis

Data analysis was started simultaneously with data collection. Two authors (FSH, JJN) independently used content analysis with MAXQDA software to analyze the data. Content analysis method consists of five steps including: familiarization, inducing themes, coding, elaboration, and interpretation and checking. All interviews were recorded and transcribed verbatim. The texts were reviewed several times for obtaining a general overview. Then, the texts were studied line by line in order to provide a comprehensive view of each line. At first, data coded was done by two researchers which indicates analysis units. Analysis units are the answers for questions. Semantic units were extracted from the main concepts of analysis units and each of them was provided with a specific code. The codes were compared with each other and an index of main and subsidiary codes was made. In coding of the second level, main and subsidiary codes were recited and those codes with similar meanings were categorized. In case of any dissimilarity, after discussion and reaching an agreement, the final code was determined.

### Trustworthiness

During the study, four factors of dependability, credibility, confirmability, and transferability which have been provided by Lincoln and Guba [21], were used to ensure the trustworthiness of data, which is the equivalent of validity and reliability in the quantitative studies. These factors are the standards of scientific rigor in qualitative studies. To ensure the credibility of data, member checking method was used. For this purpose, the interview transcripts and the drafted themes were given to the related participants to review the results and verify them. Also, we performed a sampling with a maximum variation of age, sex, and socio-economic factors. In order to ensure transferability, we provided necessary explanation regarding the study context, data collection and analysis. Also, we sought the opinion of three diabetic patients with similar characteristics to the participants who did not participate in the study to judge the similarity of the results to their own experiences. To approve the dependability of the results, the data were examined by four prominent researchers in this field who were not involved in this study. To increase confirmability, while trying to avoid bias by researchers, all authors of the paper were involved in the process of analyzing and coding and all of them were present at the meetings and expressed their views.

### Ethics

This paper is extracted from the research project approved by the Research Center of Social Factors in Health in Kerman University of Medical Sciences with project number 930482. Ethical approval for this study was obtained from Ethics Committee of the Kerman University of Medical Sciences [The code of Ethics: IR.KMU.REC.1393.654]. Written informed consent was taken from all the participants. All methods were carried out per relevant guidelines and regulations. All participants were assured that their comments would be confidential and that no identifying information would be published.

## Results

Reasons for non-participation in the self-care training program were categorized into five themes and 14 sub-themes. The relevant themes and sub-themes are depicted in Table 2.

**Table 2:** Reasons for diabetic patients’ non-participation in self-care training program

Theme	Sub-theme
Access	Physical access
	Time access
Individual, familial and social factors	Physical abilities
	Social-familial responsibilities
Attitude and awareness	Attitude to disease
	Attitude to education
	Attitude to health
	Awareness
Motivator factors	Incentive
	Communication
	Teaching methods
Need Factors	Perceived risk
	Access to other educational resources
	Self-efficacy

### Access

#### Physical access

Patients stated that they did not attend self-care training classes due to the long distance from the location of classes and the lack of facilities such as parking and an elevator.

“Classes were held in crowded districts of the town, and they (training course organizers) expect us to be on time for their classes every session. The class was held in the most polluted part of the city. I come to control my blood sugar, I ruin my lungs.” (M12)

“Every time I come to class, and I have to look for a parking space in the neighborhood for about half an hour. Because I got parking tickets several times for parking in the wrong place, I decided not to go anymore. This place has stairs and I cannot come because I have a backache and pain in my legs. If classes are held on the ground floor, I will come. When a center does not have a parking lot and an elevator, is better to decide not to attend its classes. This is not a very high expectation; it is minimum standards that must be met.” (M30)

#### Time access

Some patients drop out of the courses due to the untimeliness of the classes or temporal interference of the classes with their work hours.

“Classes were held at the traffic peak time in the city center. It takes a long time for me to go to class and back, and I have to be in traffic for an hour or two. But it is very good if it is in non-traffic and secluded hours, under that condition I will come every day.” (M9)

### Individual, familial and social factors

#### Physical abilities

Physical problems have been stated as other obstacles to participating in self-care training classes. The patients said that due to such physical problems as a backache and leg pains, cardiovascular diseases and old age, they were not able to attend the classes.

“You know, I have a bad knee ache and it’s difficult for me to walk and sit in the class for a long time. I also have heart disease. It would be hard for me to come to the class when the weather gets hot.” (M11)

#### Social-familial responsibilities

Different responsibilities of people in family and society have prevented people from participating in and benefitting from self-care classes. Family responsibilities include responsibilities that are assigned to individuals through roles such as motherhood or fatherhood. Especially in Iranian society, women in the family have extensive responsibilities as a mother, daughter or daughter-in-law of a family to care for family members, which sometimes prevents them from taking care of themselves and participating in self-care education programs. Social responsibilities also include the responsibilities that a person has as an employee or employee.

“I am a mother, and I have a range of roles in my family such as cooking, washing the dishes, etc. I had to pick up my son after school then. Some of my class times coincided with the days on which I had to take care of my mother-in-law and there was no one to take care of her instead of me.” (M8)

“I'm not allowed to leave the workplace. People like me who are clerks may be able to take time off from work to attend only two or three sessions out of 12.” (M19)

### Attitude and awareness

#### Attitude to disease

Some participants deem diabetes as a disease without serious effects and implications, or they consider it a harsh and incurable condition. On the other hand, the attitude of participants toward this ailment impacts their non-participation in the self-care training program.

“It is also hard for me to admit I am diabetic. I usually try to hide my condition from others because others would think about me a different way.” (M25)

#### Attitude to education

The negative attitude of the participants to the effectiveness of the classes in the creation of self-care skills and management of diabetes can lead to the reduction in participation in the self-care training program.

“By the way, I do not think these classes have much effect on the formation of self-care. A course is held and after a while it will completely forgotten.” (M5)

#### Attitude to health

The level of people’s attention to health is another factor affecting their participation in these educational classes. For example People who pay more attention to their health, take part in classes more. Because attitudes toward health are reflected in the actions, judgments, and experiences of individuals about the factors that affect their physical and mental well-being.

“Following up and taking part in classes bear no results for us. We are too old. I think those who attend regular training classes really care about their health.” (M28)

#### Awareness

One of the most effective factors leading to the reduction in participation in self-care training programs is the lack of patients’ awareness about diabetes and its side effects and the necessity for self-care education. They believed that keeping people cognizant through social networking applications of the necessity for taking part in educational classes and the schedule of these classes helps to increase this behavior.

“Promulgation is not just posting banners, etc., or the notice on the door of the diabetes unit director saying ‘take care of the foot’. The reason why most people do not attend these classes and do not care about them, is the lack of their awareness of the complications of diabetes. You should try much more to make people aware of diabetes and give the necessary warnings about the consequences and dangers of this disease in order to reduce the indifference of the people towards this disease and to encourage them to follow their progress. I, myself do not know enough about the complications of diabetes and that is why I did not take diabetes so seriously so I didn’t want to try to control it and to attend these classes.” (M30)

### Motivators

#### Incentive

Encouraging factors effective in attracting people to training classes. Giving rewards such as free use of educational books and CDs for continuous and regular participation in training courses, the effectiveness of instruction, and getting tangible results are some rewarding factors encouraging patients to attend the classes regularly.

“It wasn't such a class atmosphere that encourages me to continue taking part in classes. If the environment was in such a way that it would be possible to use books or educational films for free, or the results of the class were tangible, I had more incentive to participate.” (M14)

#### Communication

The weak communication between the patient and the instructor and weakness in the communicative skills of the educational personnel were the reasons for non-participation in self-care training classes. From the participants’ point of view, this communication weakness is due to reasons such as poor communication skills of the staff or their unwillingness to communicate or the incompatibility of the training with the literacy level of the participants.

“There is no proper emotional relationship between the doctor or the health center expert and the patient... If we are supposed to come to classes in which we have to tolerate an unfriendly atmosphere, then it is clear that we do not like to come. When doctors talk to patients, they sometimes look down and are unwilling to look into the patient's eyes... while the patient must feel that the doctor understands him and s/he can trust him. For example, if a patient is eager to learn more and asks a question about it, he will face a bad reaction from the doctor or the center, unfortunately, and this can cause discouragement and leaving the courses like this.” (M26)

“I have elementary education, and the woman who taught us spoke ostentatiously and I did not understand most of what she said.” (M28)

#### Teaching methods

The lack of attractive teaching methods and suitable teaching resources was another reason for diabetic patients’ non-participation in the training program. Using various attractive teaching methods and suitable teaching resources can be effective in not only helping patients learn more and enabling them, but also attracting them to participation in classes. The interviewees referred to such attractive educational paraphernalia as slides and movies, giving educational CDs, offering key educational points through SMS, and narrating the experiences of successful patients in controlling their condition as effective factors in enjoying self-care classes.

“The classes were very formal, I was not comfortable in class at all and I felt sleepy in class. I liked that educational videos or the experiences of patients who have been successful in controlling diabetes were used in the classes.” (M3)

### Need factors

#### Perceived risk

Feeling or not feeling threatened by the disease is effective in participating in self-care training schemes. This means that the more people feel threatened, the more they feel the need to participate in self-care education programs, and vice versa. Part of this perceived sense of danger comes from people’s experiences of interacting with other diabetics who have more complications.

“I thought I have just started taking metformin pills and still have a long way to insulin injection and nail bruises and blindness. My blood sugar was the lowest among the people in the class. That's why I was lazy to continue classes because I knew my disease was not acute yet and my blood sugar was at the border, so I did not take it too seriously, but now in a recent test I saw that my blood sugar has been raised up, and I decided to really follow up.” (M23)

“Because we heard that they amputated the arm or leg of someone due to diabetes, naturally we are sensitive but I don't think this will happen to me.” (M10)

#### Access to other educational resources

Direct and simpler access to other educational programs and social networks related to self-care reduces participation in self-care educational classes and brings about insufficient exploitation of such classes.

“Well, if I have a question about taking care of myself, I’ll search the Web and find my answer, or call to a consultant. Nowadays It's so easy to connect to the Internet and answer any questions. We get a lot of this information through the public media, the Internet, and so on. Why pay for attending classes for no reason.” (M6)

#### Self-efficacy

Self-efficacy is the belief that a person has the ability to organize and implement the necessary actions in the situations ahead, and it can determine the way people think, behave and feel. Having sufficient information and knowledge about the management of diabetes and the creation of a feeling of efficacy was the reasons for the patients’ dropping out of self-care educational programs.

“I know for myself what steps should be taken to control diabetes. In the first few sessions, I realized that my own information was enough to control our illness. Most of the time I do not eat sugar and I treadmill for 10 minutes a day, and I follow my diet. So I do not need to attend these classes.” (M27)

## Discussion

The qualitative study was done to identify the reasons for diabetic patients’ non-participation in self-care training programs. As a result of analyzing the findings, the five main themes including access; individual, familial and social factors; attitude and awareness; motivators and need factors were extracted.

Access was the first obstacle to patients’ participation in self-care training programs, which include sub-codes such as physical access and time access. In another study, a lack of enough and suitable space for education is reported as a main problems related to participation in self-care courses [22]. Most of the audience who refused to take part in classes due to weak local access was from the old-age group. Since the elderly prevails among the patients and audiences of the classes, it is thus crucial to facilitate local access for the participants in the educational courses. Using locations with a suitable structure and holding classes on the ground floor, especially for the elderly, must be taken into consideration. Besides, in-person education at home or on the phone and distance education through mass media and cyberspace are among the reliable methods to communicate information to patients and their family members and to solve spatial problems.

Individual, social and familial factors are other obstacles on the way to participating in the self-care training schemes. In line with the current study, previous studies have suggested hectic work and business and difficulty in getting time off work as social obstacles to the training and self-care of diabetic patients [23, 24]. Previous studies showed that patients who are unemployed or retired are more likely to attend in self-management programs than those who work full or part-time [10, 25]. Therefore, having flexible timing and holding classes in the afternoon, and making use of alternative methods of educational classes help to alleviate these obstacles. It is reported that support from fellows and family members as the facilitating factor in enabling individuals in self-care behavior in their study [26]. Thus, awareness of family members about the importance of self-care behavior and the necessity for participation in educational programs to support the patients in their participation in educational classes is crucial.

Another effective factor in the reduction in exploiting self-care educational programs among diabetic patients is related to the physical abilities of the patients. Old age and suffering from chronic diseases result in physical disabilities and problems leading to impediments to participation in educational classes. Mogre et al. have also identified old age and pain as obstacles to the implementation of self-care behavior [27]. Making use of mobile phone applications leads to an increase in self-care behavior in patients with chronic diseases [28]. Therefore, designing and using educational self-care mobile phone applications, virtual education or in-person training by healthcare providers, and teaching the patients’ family members are among the solutions for the above-mentioned challenge.

Attitude and awareness including sub-themes such as attitude to disease, attitude to education, attitude to health, and awareness were other reasons for patients’ non-participation in self-care programs. Understanding diabetes on the part of patients in terms of preserving self-care behavior is vital. Mogre et al. have also identified the false image of contracting diabetes as one of the obstacles to self-care in their study [27]. The findings of the present study identify the negative attitude toward the effectiveness of self-care behavior in controlling diabetes as one of the obstacles to the implementation of self-care behavior in patients. This result is consistent with the results of study conducted by Gucciardi et al. [10]. Part of these attitudes is the result of cultural beliefs identified in the study done by Whittermore et al. as one of the obstacles to the creation of self-care behavior [29]. It seems that unless individuals acquire a proper insight into diabetes, the possibility of participation in in-person classes will not increase. This, in turn, needs a clear explication of the importance of the disease and its dangerous side-effects on the part of healthcare providers, instructors, health media, and diabetes associations.

Another effective factor in the exploitation of self-care educational programs is patients’ little awareness of the importance of educational schemes, and the time and location of the educational classes. Concurrent with the findings of the present study, another study identify a paucity of proper promulgation about educational programs and lack of information on the part of service providers about the importance of the patients’ participating in such programs as fundamental problems of diabetic patients with self-care [30]. Studies show that in developed countries patients have higher expectations and more information should be provided for patient participation. These studies found that centers that provide patients with information on when and where to go, where to park, what to bring, whom they will see, and what to expect, in addition to providing a reminder call prior to the appointment, dramatically reduce initial non-attendance rate [31].

The lack of motivators was another reason for patients’ non-participation in self-care programs. One study showed that the absence of heartening instruments by healthcare providers as motivational obstacles effective on participation in educational programs [30]. Monitoring and following up with patients after participating in educational classes via telephone or in-person contribute to their more success in controlling their ailment and more participation in educational classes. Providing awards and prizes for those who attend the classes regularly as offered by patients. Weak connections and mutual misunderstandings between patients, healthcare providers, and instructors also contribute to individuals’ disinterest in taking part in educational classes. The findings of various studies showed that the relationship between the doctor and the patient affected the implementation and following up self-care behavior [17, 32]. Therefore, teaching and enhancing the communicative skills of service providers and instructors are of the exigencies of holding educational courses. Although training is one of the most effective ways to enhance self-care conduct, weakness in contents and unsuitable teaching techniques can hinder the use of the educational programs, which are also mentioned by the patients in the present study. In another study, unsuitable teaching content is said to be one reason for the disinterest in such schemes [24]. Using supplementary books and reflecting the experience of cured patients by themselves were suggested by the interviewees. Surveys on patients’ awareness of diabetes and favorite teaching methods can lead to better planning and increasing the attractiveness of the classes. Schafer et al. have recommended using the private than group education and adopting a multidimensional rather than presentational method because they are more effective on self-care behavior [17]. Therefore, enjoying such various teaching methods as educational technology, multimedia resources, and sending educational short messages in designing self-care educational schemes are suggested.

A feeling of not needing educational programs is an effective factor in non-participation in self-care programs. The present findings introduce ignorance of the danger of diabetes on the part of patients as another obstacle to the implementation of self-care behavior, which confirms the results of previous studies [10, 33]. Thus, building awareness about the dangers of diabetes and its side effects in patients by healthcare providers, mass media, and affiliated associations seems all-important.

Inaccessibility of educational information through other resources such as the media, the Internet and tutorial booklets is another obstacle to participation in educational programs. Various studies have shown that using suitable educational apparatus (laptop, DVD, and PowerPoint), phone tracking, mobile virtual system, and email is effective in teaching self-care to cardiac patients [34, 35]. In the status quo that access to teaching and learning resources in society seems to have reached wide areas, it is better to move toward new educational methods, especially since these methods lead to the economization of people’s time and educational expenses. Despite the advantages of these educational methods, unknown and adverse information given by the media will result in distrust of therapeutic programs [36]. As a result, surveillance and giving, the necessary instructions to implement these educational methods should be considered by healthcare planners and decision-makers.

Self-efficacy is the trust put in one’s abilities by themselves to follow up behavior and is a vital prerequisite to behavior change [37]. In the present study, the patients did not continue taking part in the educational classes after they had acquired the ability to handle their disease and created a feeling of self-efficacy. In the study done by Gucciardi, patients’ self-confidence in the cognition of their abilities to manage diabetes was also identified as an obstacle to participation in self-care educational programs [10]. Therefore, regarding the fact that patients do not attend the educational classes following the creation of self-efficacy, phone tracking by providers to monitor self-care behavior in such patients can contribute to the better implementation of this salubrious behavior.

One of the limitations of this study was a lack of collaboration on the part of the health center officials and those who participated in the study as well, who were briefed to cooperate by offering explanations about the advantages and importance of the study. Individuals’ disinterest in going to the health center for the interview was another limitation that was alleviated by going to people’s homes and workplaces. We excluded diabetic patients who participated in the self-care program, therefore, we could not examine the factors influencing people’s participation in self-care programs.

Recommendations for further research include economic evaluation studies of training programs for diabetic patient, longitudinal studies that examine the impact of training programs on quality of life and costs of diabetic patients.

## Conclusion

This study concludes that there are many barriers against the participation of diabetic patients in self-care programs that should be facilitated. Lack of suitable facilities, local and temporal access, high-quality education through attractive teaching methods and contents, getting support from family members and diabetic patients’ friends, virtual and distant training for patients with physical disabilities, communicative skills of healthcare professionals and health service providers were as barriers to participation.

## Data Availability

All data are available on reasonable request to the first author.
